# The metastasis-inducing protein AGR2 is *O*-glycosylated upon secretion from mammary epithelial cells

**DOI:** 10.1007/s11010-015-2502-3

**Published:** 2015-07-14

**Authors:** Christopher Clarke, Philip Rudland, Roger Barraclough

**Affiliations:** Institute of Integrative Biology, University of Liverpool, Biosciences Building, Crown Street, Liverpool, L69 7ZB UK

**Keywords:** AGR2, Glycosylation, Secretion, Adhesion

## Abstract

AGR2 is overexpressed in multiple cancers, particularly those arising from breast and prostate tissues, and higher levels of AGR2 are associated with earlier patient death. Although AGR2 is normally resident within the endoplasmic reticulum, the protein has been found in the extracellular space in several model systems. However, it has never been expressly demonstrated that this extracellular form of the protein is secreted and does not just accumulate in the extracellular space as a result of cell lysis. We show in this paper that AGR2 protein is secreted by both human and rat mammary epithelial cells in culture. Furthermore, this secreted form of AGR2 becomes *O*-glycosylated, with no detectable presence of *N*-glycosylation. Importantly, this post-translationally modified AGR2 is only detected in the conditioned medium from non-leaky cells, suggesting that membrane integrity must be maintained to allow AGR2 glycosylation. The results suggest a possible role for *O*-glycosylation in modulating the extracellular functions of AGR2.

## Introduction

Anterior gradient protein 2 (AGR2) is a member of the protein disulphide isomerase family of endoplasmic reticulum (ER) chaperones [1], and its major role to date appears to be in promoting the secretion of several mucin glycoproteins, including MUC1 [2], MUC2 [3, 4], MUC4 [5], MUC5B and MUC5AC [6, 7]. It also appears to promote cell differentiation and, as such, is involved in the development of lung [7], breast [8], liver [9] and gut [10] tissues.

Expression of AGR2 is induced by physiological stress [11], ER stress [3, 6, 11–14], and is strongly oestrogen- and androgen-responsive, particularly in breast and prostate tissue [15–18]. For this reason, overexpression of AGR2 is often reported in a number of cancers, particularly in those arising from the breast, prostate, ovary and pancreas. In these cancers, higher expression levels of AGR2 generally correlate with decreased patient survival time [5, 19–24]. The reduced survival time is thought to be caused by an increase in the rate of metastasis of AGR2-expressing cancers, as a number of studies have shown an increase in AGR2 expression in metastatic cells relative to their primary tumours [16, 25–31] and importantly, AGR2 was able to induce metastasis of a benign rat mammary cell line when cells overexpressing AGR2 were injected into syngeneic rats [16].

AGR2 is largely tethered to the ER through its C-terminal ER-retention sequence [4, 14, 32–34], but several studies have reported the presence of AGR2 in the extracellular compartment [35–38], although it has never been demonstrated that this AGR2 is not just simply released as a result of cell death and lysis. However, in vivo, AGR2 has been detected in both human colonic mucus [35] and murine intestinal mucus [35–37]. This suggests that AGR2 may have an extracellular role, perhaps in a similar way to nAG, the newt homologue of AGR2, which binds to the cell surface receptor Prod1 and can induce limb regeneration in a salamander model [39, 40]. As further evidence of an extracellular role for AGR2, a recent study showed that the addition of extracellular recombinant AGR2 to pancreatic cancer cells promoted their growth, migration and invasion by signalling through the C4.4a cell surface receptor [41]. Interestingly, C4.4a is a structural homologue of the urokinase-type plasminogen activator receptor (uPAR), which, along with CD59, is the most closely related human homologue of Prod1 [42]. Extracellularly-added recombinant AGR2 has also been shown to promote adhesion of rat mammary epithelial cells [16, 43] and the migration and tube formation of human umbilical vein endothelial cells (HUVEC) [38], indicating a possible role for extracellular AGR2 in the promotion of angiogenesis. Changes in both cell adhesion and induction of angiogenesis may be important for a pro-metastatic phenotype.

The prevalence of AGR2 overexpression in several tumour types and its correlation with patient survival has engendered much interest in the use of AGR2 as a serum or urine biomarker for disease detection [29, 44–47]. Further understanding of the nature of secreted AGR2 may be important in maximising the accuracy and sensitivity of such tests, and may also shed light on the extracellular functions of AGR2. We show here that AGR2 is *O*-glycosylated upon secretion from human and rat cell lines, and that this form of the protein is released from healthy cells and not as a result of cell lysis.

## Materials and methods

### Cell culture and transfection

The oestrogen receptor-positive MCF7A human mammary epithelial cell line was grown in Dulbecco’s Modified Eagle’s Medium (DMEM, Life Technologies) including non-essential amino acids (NEAA) and supplemented with 10 % (v/v) foetal bovine serum (FBS), 4 mM l-glutamine and 10 µg/mL insulin (Sigma). Rama 37 rat mammary benign epithelial tumour cells [48] were grown in DMEM including NEAA and supplemented with 5 % (v/v) FBS, 4 mM l-glutamine, 10 ng/mL insulin and 10 ng/mL hydrocortisone (Sigma). Cells were transfected with the PiggyBac EF1α-IRES-neomycin plasmid vector (Systems Biosciences, vector only), or the same vector containing a human AGR2 cDNA, using FuGENE6 transfection reagent, according to the manufacturer’s protocol (Promega). Transfected cells were maintained in normal growth medium supplemented with 1 mg/mL G418 (Melford). Clonal cell lines were created by serial dilution of transfected cells down to a single cell in a known volume and growing these for several weeks until confluent cultures were obtained.

### Collection of conditioned medium

Cells were grown to 30–40 % confluence, washed four times in phosphate-buffered saline (PBS) supplemented with 900 µM CaCl_2_ and 500 µM MgCl_2_, in order to remove the maximum amount of FBS whilst limiting cell detachment. These cells were then incubated in Opti-MEM (Minimum Essential Medium) serum-free medium (Life Technologies) supplemented with a final concentration of 25 mM glucose, 1.8 mM CaCl_2_, 4 % (v/v) non-essential amino acids (NEAA, Life Technologies) and 10 ng/mL hydrocortisone, in order to make this serum-free medium as close to the formulation of normal culture medium as possible. After 24 h, medium was collected, centrifuged at 1000×*g* at 4 °C and concentrated using an Amicon ultra centrifugal filter (Millipore).

### Western blotting

Whole cell lysates were prepared from cells grown to 70–80 % confluence and lysed in RIPA buffer [50 mM Tris–HCl, pH 6.8, 150 mM NaCl, 2 mM EDTA, 1 % (v/v) NP-40, 0.5 % (w/v) sodium deoxycholate, 0.1 % (w/v) SDS and complete protease inhibitor cocktail (Roche)]. Secreted protein samples were obtained as described above. Samples were subjected to SDS-PAGE, transferred to PVDF membrane (Millipore) and probed with a mouse monoclonal antibody specific for AGR2 (Millipore, MABC48) or a polyclonal rabbit anti-LDHA (Cell Signaling Technology, #2012) serum. Horseradish peroxidase-coupled secondary antibodies were obtained from Dako.

Protein molecular weights were estimated by plotting a standard curve of molecular weight and relative migration distance for known protein standards, and calculating the molecular weights of different forms of AGR2 using their relative migration distance.

### Enzymatic deglycosylation of proteins

All enzymes and reagents were acquired from New England Biolabs. Cell lysates and samples of conditioned medium were first denatured by incubation with 0.5 % (w/v) SDS, 40 mM DTT and subsequent heating at 98 °C for 5 min. Samples were then subjected to treatment with either PNGase F, *O*-glycosidase/neuraminidase mix or protein deglycosylation mix (consisting of PNGase F, *O*-glycosidase, neuraminidase, β1-4 galactosidase and β-*N*-acetylglucosaminidase) for 4 h at 37 °C, as per the manufacturer’s instructions.

## Results

### AGR2 is secreted in a higher molecular weight form from cultured rat and human mammary cells

**Fig. 1:**
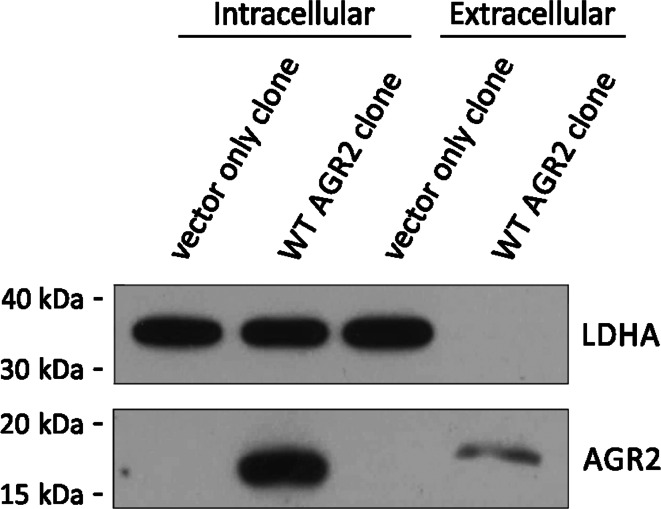
A higher molecular weight form of AGR2 is released into the medium of AGR2-expressing rat mammary epithelial tumour cells. Conditioned medium was collected from vector only-expressing or WT AGR2-expressing Rama 37 cells and analysed for the presence of AGR2 and lactate dehydrogenase (LDHA) by Western blot. The presence of LDHA in the conditioned medium was used as an indication of the contamination of the secreted protein pool by intracellular proteins [64]. Extracellular AGR2 was 0.90 (SD ± 0.12) kDa larger than intracellular AGR2 on average (*n* = 3)

A number of studies have reported that AGR2 is secreted, both from cultured cells [17, 25, 49] and into intestinal mucus in mice [35]. In the present experiments, AGR2 was recovered from the culture medium of Rama 37 cells expressing wild-type (WT) AGR2 protein and displayed an apparent molecular weight 0.90 (SD ± 0.12) kDa higher than that of intracellular AGR2 (Fig. 1). This mass difference in extracellular AGR2 shows that its presence in the culture medium is not due to intracellular AGR2 released from lysed or leaky cells, and this is further confirmed by the lack of detectable levels in the culture medium of AGR2-expressing Rama 37 cells of the intracellular marker protein, lactate dehydrogenase A (LDHA).

**Fig. 2:**
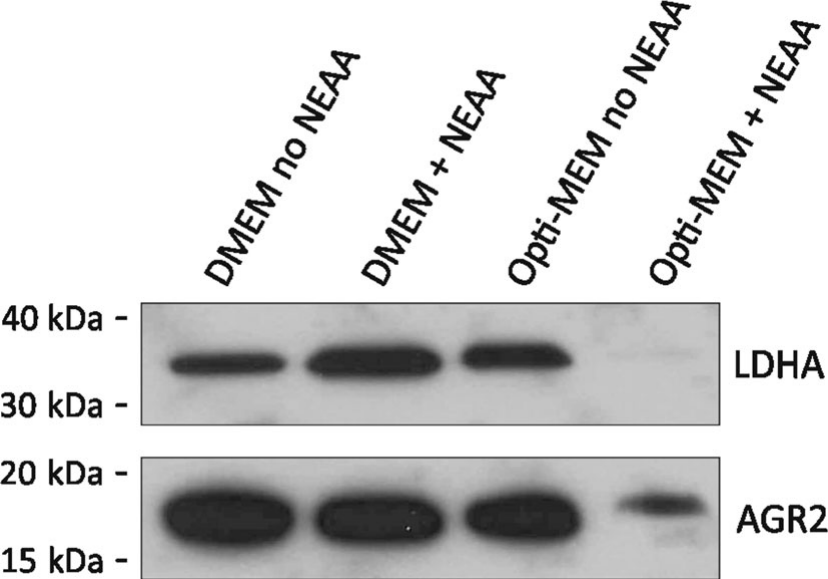
In human mammary epithelial cells, higher molecular weight AGR2 is only released from non-leaky cells. MCF7A cells were incubated for 24 h in serum-free DMEM or serum-free Opti-MEM medium, with and without non-essential amino acids (NEAA). Conditioned medium was collected and probed for AGR2 and LDHA by Western blot. The presence of LDHA in the conditioned medium was used as an indication of the contamination of the secreted protein pool by intracellular proteins under each serum-free condition. Extracellular AGR2 was 0.93 (SD ± 0.09) kDa larger than intracellular AGR2 on average (*n* = 3)

To ensure that this release of higher molecular weight AGR2 was not restricted to transfected rat mammary cells, the release of AGR2 from AGR2-expressing human MCF7A cells [16] was investigated (Fig. 2). Furthermore, to investigate further whether this higher molecular weight form is actively released from cells rather than released by cell lysis, MCF7A cells were incubated in increasingly nutrient-poor media, and the presence of AGR2 in these conditioned media was monitored by Western blot (Fig. 2).

All incubation media were serum-free (see “Materials and methods” section), and cells incubated in either DMEM with or without non-essential amino acids (NEAA), or with Opti-MEM medium without NEAA released large amounts of lower molecular weight AGR2 into the culture medium, but there was no detectable higher molecular weight AGR2 under any of these conditions. Furthermore, cells incubated in these media also released high amounts of LDHA, suggesting that AGR2 is released from these cells as a result of cell lysis. Conversely, cells incubated in Opti-MEM medium containing NEAA released a form of AGR2 with apparent molecular weight 0.93 (SD ± 0.09) kDa larger than intracellular AGR2, but barely detectable levels of LDHA. These data suggest that the higher molecular weight form of AGR2 is released only from intact cells that do not leak intracellular proteins.

### Secreted AGR2 contains *O*-linked, but not *N*-linked, glycans

**Fig. 3:**
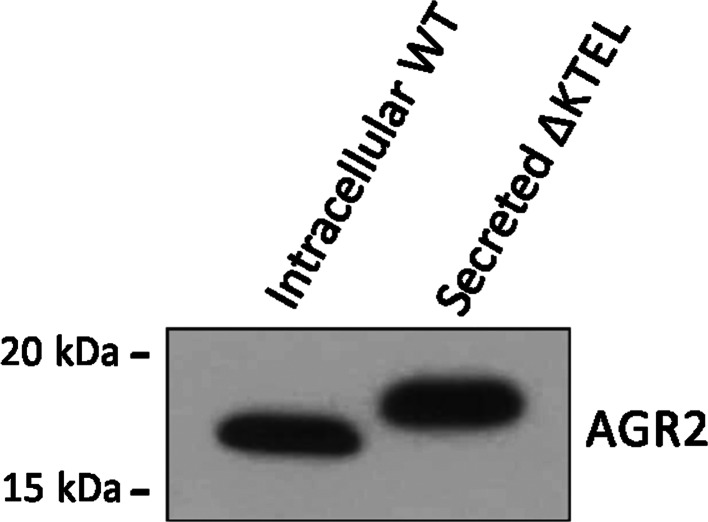
A highly secreted mutant form of AGR2 is also released in a high molecular weight form from rat mammary tumour cells. Rama 37 cells were engineered to express AGR2 devoid of the C-terminal KTEL ER-retention sequence in order to increase its secretion. Note that, as ΔKTEL AGR2 is highly secreted, intracellular levels are almost undetectable and thus expression of intracellular WT AGR2 is shown for size comparison. Extracellular AGR2 was 0.94 (SD ± 0.09) kDa larger than intracellular AGR2 on average (*n* = 3)

Given that only small amounts of AGR2 protein are secreted from both Rama 37 and MCF7A cells, we created a Rama 37 cell line expressing a mutant form of AGR2 lacking the C-terminal ER-retention sequence (ΔKTEL AGR2), leading to increased secretion of AGR2, and thus facilitating the determination of the increase in molecular weight of secreted AGR2. As expected, AGR2 was secreted in higher amounts from ΔKTEL AGR2-expressing cells but, interestingly, secreted ΔKTEL AGR2 displayed the same sized shift in apparent molecular weight compared to intracellular WT AGR2 (0.94 (SD ± 0.09) kDa, Fig. 3) as did secreted WT AGR2 (Fig. 1), despite the KTEL deletion rendering the intracellular protein apparently 0.87 (SD ± 0.01) kDa smaller than intracellular WT AGR2 (measured by polyacrylamide gel electrophoresis, data not shown).

**Fig. 4:**
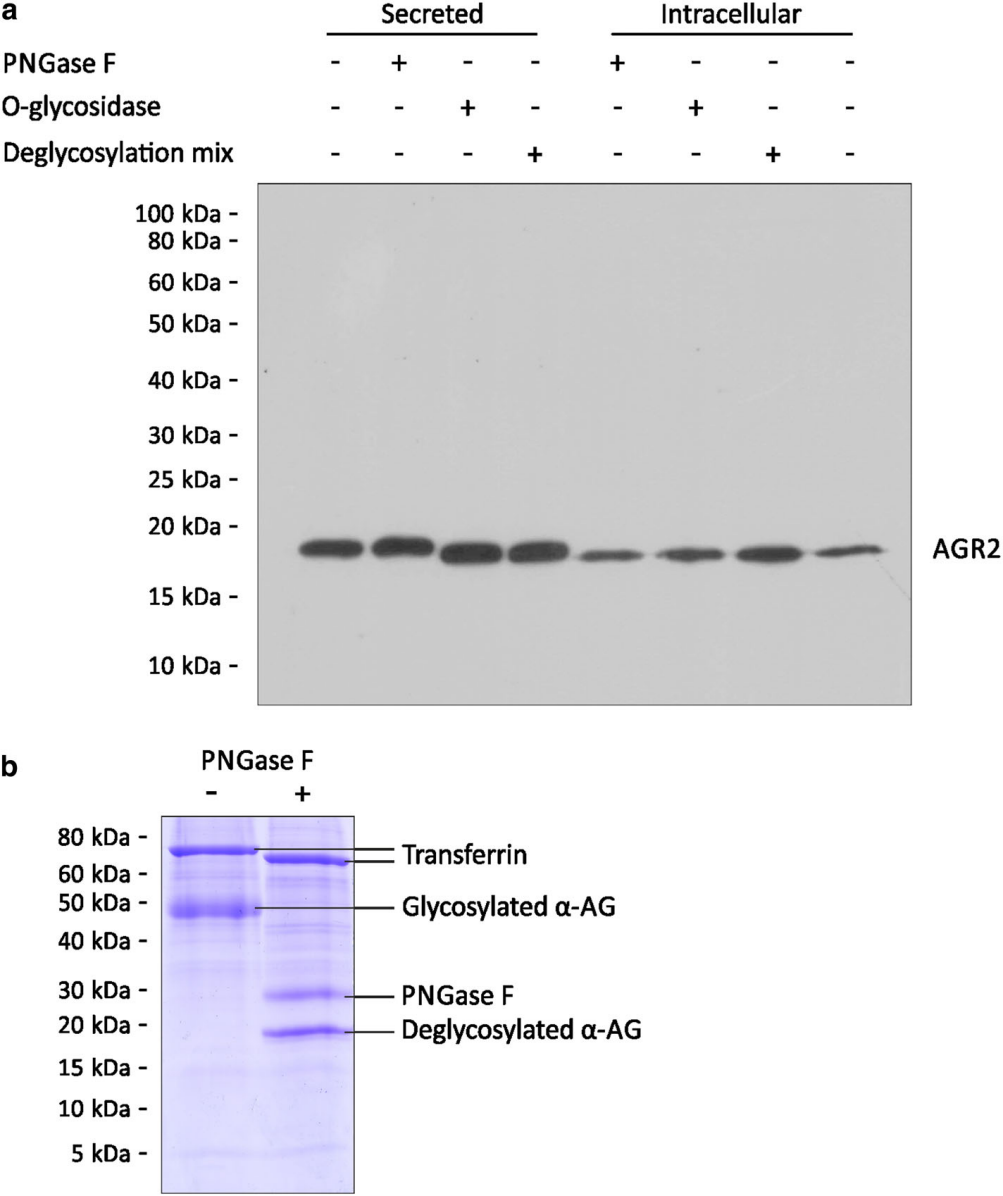
Treatment with *O*-glycosidase reduces the molecular weight of secreted AGR2. **a** Conditioned medium from ΔKTEL AGR2-expressing cells (secreted AGR2) and whole cell lysate from WT AGR2-expressing cells (intracellular AGR2) were treated with the indicated deglycosylation enzymes for 4 h at 37 °C, as per the manufacturer’s instructions. Treated samples were subjected to Western blot and probed for AGR2. PNGase F is an *N*-glycosidase. *O*-glycosidase-treated samples were simultaneously treated with neuraminidase, as the presence of terminal sialic acid residues blocks the activity of *O*-glycosidase [65]. Deglycosylation mix consists of PNGase F, *O*-glycosidase, neuraminidase, β1-4 galactosidase and β-*N*-acetylglucosaminidase. Untreated extracellular AGR2 was 0.80 (SD ± 0.04) kDa larger than intracellular AGR2 on average (*n* = 3), and extracellular AGR2 treated with *O*-glycosidase or deglycosylation mix was 0.27 (SD ± 0.04) kDa larger than intracellular AGR2 on average (*n* = 3). **b** To ensure that PNGase F was active in the presence of culture medium components, α-acid glycoprotein (α-AG) was added to conditioned medium and treated with PNGase as in** a**. Reaction mixtures were run on an SDS-PAGE gel and stained with Coomassie blue

As the majority of secreted proteins are glycosylated [50], it is likely that the cause of the increase in apparent molecular weight of secreted AGR2 was one or more glycosylation events. Indeed, secreted ΔKTEL AGR2 (but not intracellular WT AGR2) reacted weakly with periodic acid-Schiff stain, a glycoprotein stain (data not shown). Therefore, we investigated the nature of the probable glycosylation using specific deglycosylating enzymes (Fig. 4).

Conditioned medium from ΔKTEL AGR2-expressing cells treated with the *N*-glycosidase PNGase F showed no change in the apparent molecular weight of AGR2, remaining 0.80 (SD ± 0.04) kDa larger than intracellular AGR2, both with and without PNGase F treatment (Fig. 4a). Under these same reaction conditions, however, PNGase F reduced the apparent molecular weight of α-acid glycoprotein by approximately 30 kDa, indicating that the enzyme was active in the conditioned medium (Fig. 4b). Treatment of ΔKTEL AGR2-conditioned medium with either *O*-glycosidase (and neuraminidase) or a commercial mix of deglycosylation enzymes, consisting of PNGase F, *O*-glycosidase, neuraminidase, β1-4 galactosidase and β-*N*-acetylglucosaminidase, reduced the apparent molecular weight of secreted AGR2 by 0.53 (SD ± 0.04) kDa, but not back down to the apparent molecular weight of intracellular AGR2. Deglycosylation enzymes had no effect on the size of intracellular AGR2 (Fig. 4a). These experiments indicate that the secreted form of AGR2 does not contain any detectable *N*-glycosylation sites, but contains at least one digestible *O*-glycosylation site.

## Discussion

It is shown here for the first time that AGR2 secreted from both rat and human mammary epithelial cells becomes *O*-glycosylated. While it was possible to distinguish that secreted AGR2 was *O*-glycosylated, but not *N*-glycosylated, it is clear that neither treatment with *O*-glycosidase/neuraminidase nor the deglycosylation enzyme cocktail (Fig. 4) fully reduced the apparent molecular weight of the secreted AGR2 to that of the unmodified intracellular AGR2. This likely reflects the presence of glycan structures not digestible by the enzymes used herein, but while secreted AGR2 is not phosphorylated (data not shown), we cannot totally rule out the presence of some other, less common, post-translational modification. In addition, the similar-sized shift in apparent molecular weight observed between secreted WT and ΔKTEL AGR2 (Figs. 1, 3), despite the smaller size of the ΔKTEL polypeptide chain, implies that the secreted ΔKTEL AGR2 may be more heavily glycosylated than WT protein. The presence of a KTEL sequence has been previously shown to alter the make-up of *O*-linked glycans, and probably relates to the differences in transit time of the protein from ER to Golgi and to the extracellular space [51]. Due to the limitations of the SDS polyacrylamide gel electrophoresis technique, it was not possible to draw further conclusions about the detailed nature of the *O*-linked modifications from size differences of the observed bands.

**Fig. 5:**
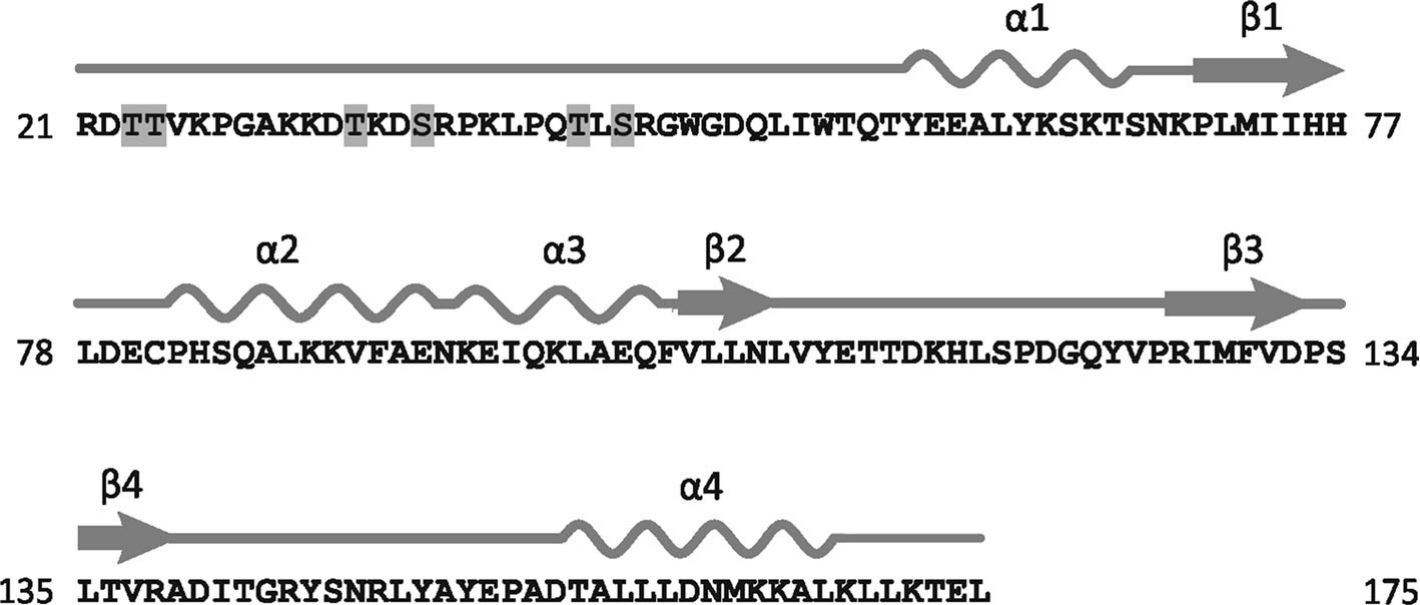
Predicted* O*-glycosylation sites on secreted AGR2. Amino acids predicted as possible sites of *O*-glycosylation by the NetOGlyc 4.0 server [52] are shaded in grey boxes. Secondary structure elements are shown above the primary sequence. α:α-helix. β:β-sheet. Figure adapted from [43]

Possible sites of these *O*-glycosylated residue(s) in AGR2 are shown in Fig. 5, based on prediction by the NetOGlyc 4.0 server [52]. All of these predicted sites lie within the unstructured N-terminal region of AGR2, which we described previously [43], consistent with a recent bioinformatics finding that *O*-glycosylation sites are located preferentially in unstructured protein regions, whereas the opposite is true for *N*-glycosylation [53].

AGR2 is an ER-resident chaperone protein and other such chaperones have also been reported to be secreted [54–57]. Notably, the AGR2-related protein disulphide isomerase, ERp44, which, like AGR2, contains a divergent CXXS-active site, is *O*-glycosylated upon secretion [57]. It is possible therefore that *O*-glycosylation of normally ER-resident chaperones by the Golgi body is a more general phenomenon, rather than an AGR2-specific event, and may stem from ‘leakiness’ of the KDEL-dependent retrograde transport of proteins from Golgi to ER [51]. Furthermore, although most secreted proteins passing through the Golgi are glycosylated, there are several examples of non-glycosylated secreted proteins, notably insulin [58], serum albumin [59] and elastin [60]. This suggests that glycosylation of secreted ER proteins may have functional consequences, rather than being the result of some default programme of glycosylation for any protein passing through the Golgi.

It is not yet known how the presence of the *O*-linked glycosylation might affect the biological activity of AGR2. The protein plays a role in cell adhesion [16, 43], although it should be noted that these experiments were performed using bacterially-derived (i.e. non-glycosylated) AGR2, but it is interesting that *O*-glycosylation status has been reported to influence cell adhesion; for example, in pancreatic carcinoma cells, enhancement or reduction of cell adhesion depended upon the cell surface *O*-linked glycosylation state of the cells [61], and similarly, alterations in the *O*-glycosylation patterns of the transmembrane proteins α_2_β_1_ integrin and E-cadherin altered the migratory and invasive potential of pancreatic adenocarcinoma cells [62]. Loss of *O*-glycosylation sites in the secreted metastasis-inducing protein osteopontin was also shown to increase its pro-adhesive effects [63]. Thus, the degree and pattern of *O*-glycosylation in both cell surface and secreted proteins appear to play roles in cell adhesion. We have already shown that the unstructured region of AGR2, comprising amino acids 21–40, is required for AGR2-promoted cell adhesion [43], and we have shown here that it is also the possible site of *O*-linked glycosylation in AGR2 (Fig. 5). Glycosylation of extracellular AGR2 may therefore be important for AGR2-mediated cell adhesion.
